# Body Mass Index, Adherence to a Healthy Lifestyle, and Breakfast Consumption Associated with Religious Affiliation in Peruvian University Students: A Cross-Sectional Study

**DOI:** 10.3390/nu16152489

**Published:** 2024-07-31

**Authors:** Luis Lévano-Matos, Jacksaint Saintila, Norma Del Carmen Gálvez-Díaz, Yaquelin E. Calizaya-Milla

**Affiliations:** 1 Research Group for Nutrition and Lifestyle, School of Nutrition, Faculty of Health Sciences, Universidad Peruana Unión, Lima 15457, Peru; luislevano@upeu.edu.pe; 2 Research Group for Nutrition and Healthy Behaviors, School of Medicine, Faculty of Health Sciences, Universidad Señor de Sipán, Chiclayo 14001, Peru; ncarmengd@hotmail.com

**Keywords:** Adventists, breakfast, Catholicism, healthy lifestyle, obesity, Protestantism, university students

## Abstract

Background: Despite evidence suggesting a relationship between religiosity and health habits, there is a paucity of studies specifically examining this association in the context of Peruvian university students. This study compared body mass index (BMI), adherence to a healthy lifestyle, and breakfast consumption in Peruvian university students of four religious denominations: Seventh Day Adventists (SDA), Catholics, Baptists, and Evangelicals. Methods: A cross-sectional study was conducted online among 4557 students from a Peruvian university. The BMI and the frequency of breakfast consumption were evaluated, and the Diet and Healthy Lifestyle Scale (DEVS) was applied. The variables studied were associated using simple and multiple linear regression and Poisson models with robust variance. Results: Baptist (B = 0.44, 95% CI: 0.10–0.78; *p* = 0.011), Catholic (B = 0.3, 95% CI: 0.12–0.47; *p* = 0.001), and Evangelical (B = 0.32, 95% CI: 0.09 to 0.64; *p* = 0.014) students had a significantly higher BMI compared to SDA. Baptist (B = −0.2, 95% CI: −0.37–−0.05; *p* = 0.017) and Evangelical (B = −0.13, 95% CI: −0.28–−0.03; *p* = 0.012) students exhibited a lower mean score on the measure of healthy lifestyles compared to SDA students. Additionally, Baptist (PR = −0.32, 95% CI: −0.92–−0.12; *p* = 0.035) and Catholic (PR = −0.3, 95% CI: −0.99–−0.19; *p* = 0.016) students exhibited a lower probability of eating breakfast regularly compared to SDA students. Conclusions: Health professionals should consider these findings when designing and implementing health promotion programs that are culturally sensitive and respectful of the beliefs and practices of all religious groups in university settings.

## 1. Introduction

Healthy lifestyles are essential for the general health and well-being of university students [1]. Maintaining a healthy lifestyle, which includes aspects such as a balanced diet, regular physical activity, water consumption, and adequate exposure to sunlight, is essential to optimize physical and mental conditions [2]. On the other hand, breakfast provides the energy and nutrients needed to start the day and has been associated with improved academic and cognitive performance [3]. This is because students who eat breakfast regularly tend to have better academic results and a greater ability to concentrate during classes [1]. The importance of these healthy habits is further underscored by the fact that university students are faced with a multitude of stressors and academic demands [4]. However, during the transition to university life, students experience numerous changes in their daily routines, which can affect their living habits [5]. Indeed, academic, social, and personal pressures can lead to the adoption of unhealthy behaviors, including the omission of breakfast, consumption of nutritionally deficient foods, and a sedentary lifestyle [2]. These behaviors not only affect academic performance but can also have long-term consequences for physical and mental health, increasing the risk of developing cardiovascular diseases, academic stress, anxiety, and depression [6]. Consequently, nutritionists/dieticians and other health professionals would find it beneficial to encourage students to adopt healthy lifestyles as a form of intervention.

### 1.1. Religious Affiliation and Health

In recent decades, the proposition that religion could exert a considerable influence on health outcomes has attracted considerable skepticism from many practitioners and researchers [7]. However, in recent years, the connection between religion and health has gained greater recognition, thanks to a growing body of rigorous social and epidemiological research [7]. Numerous studies have shown that participation in religious activities and adherence to spiritual beliefs are associated with better health outcomes and a lower risk of death [8,9]. For example, individuals who regularly attend religious services have been shown to have greater longevity and a lower mortality rate [9]. Indeed, attendance at religious services was found to be associated with a reduced risk of death from despair (related to drugs, alcohol, and suicide) among healthcare professionals [8]. Furthermore, the social and emotional support provided by religious communities can play an important role in promoting optimal health. This is evidenced by the association between social isolation and loneliness and an elevated risk of mortality [10,11].

Additionally, research has investigated the potential of religion to help individuals cope with stressful situations [12]. Religious beliefs can provide a framework to make sense of life and cope with adversity, which can lead to better emotional and psychological adjustment [13]. People with strong spiritual beliefs and religious practices have been found to have a greater ability to relax in the face of serious illnesses such as COVID-19 [14]. In addition, religious communities often foster social cohesion and mutual support, which can become a safety net for their members in times of need [15]. This community support not only provides practical help, such as care and financial support, but also a sense of belonging and purpose that is essential for mental and emotional health [16].

### 1.2. Religious Affiliation and BMI

Religious affiliation as a social determinant of health can have a significant influence on the prevalence of overweight and obesity [17]. Various religions promote different healthy lifestyles, such as healthy dietary practices and regular physical activity, which may affect the body composition of their adherents [17]. For example, members of the SDA Church tend to have a lower BMI than non-SDA members [18]. Similarly, it is suggested that SDA tend to have a lower risk of cardiovascular disease, probably due to their diet, which encourages vegetarianism [19]. On the other hand, a study found that among various Christian denominations, Baptists had a higher prevalence of obesity than other religious groups [7].

However, some studies report that the association between high BMI and Evangelical, mainline Protestant, or other Christian religions remains non-significant after controlling for demographics and health behaviors [20]. These findings are useful in understanding the relationship between religious affiliation and obesity in some Christian groups, but it is limited to addressing this relationship in the context of Peruvian university students, a demographic group that faces unique health and behavioral challenges [17]. Individuals who are active in religious communities may benefit from social support networks that encourage healthy behaviors, which in turn may promote a healthy weight [21]. However, it is not known whether, in university students, these networks may exert a similar influence due to differences in the social dynamics and academic pressures inherent in university life. This study seeks to explore the association between religious affiliation and BMI and other health indicators among university students, providing valuable information that could help health professionals and educational institutions develop culturally sensitive intervention strategies.

### 1.3. Religious Affiliation and Healthy Lifestyles

Healthy lifestyles are an integral part of the teachings of many religious denominations. In particular, some religions adopt specific dietary rules and teach that the body is a temple of the Holy Spirit, motivating their followers to maintain a healthy lifestyle [22]. For example, the SDA Church and the Church of Jesus Christ of Latter-day Saints (Mormons) are known for their emphasis on dietary practices that promote health and well-being, such as vegetarian diets that encourage the consumption of foods such as fruits, vegetables, and whole grains, and the low consumption of saturated fats [23]. In addition, SDA is known for its philosophy of health based on healthy living, which includes avoiding alcohol and tobacco and promoting regular physical activity [24]. The SDA community also emphasizes proper rest and stress reduction, factors that contribute to better overall health [19]. These norms are designed to improve not only physical, but also mental and spiritual well-being.

SDA, Mormons, and other religious groups that follow strict dietary guidelines have been shown to have healthier diets, better physical health, and greater longevity than the general population [25]. In addition, people with high levels of religiosity tend to have healthier eating habits, opting for choices such as fruits and vegetables and less calorie-dense foods [26]. The avoidance of health-risk behaviors represents an additional mechanism through which the association between religiosity and better physical health outcomes may be explained. The promotion of healthy eating practices by certain religions serves as a protective factor in the long-term prevention of diet-related diseases [27]. These practices include not only dietary choices but also the promotion of an active lifestyle and community support that reinforces these positive behaviors.

In a context where lifestyles, breakfast consumption, and BMI can significantly influence long-term health, it is necessary to explore the impact of religious beliefs and practices on these behaviors in a particularly vulnerable demographic group, such as university students. Although some religions promote teachings that encourage abstinence from harmful substances and the adoption of healthy diets, the majority of studies investigating these associations have been conducted in developed countries and general populations. Consequently, there is a paucity of literature that examines how these relationships manifest themselves in university contexts and in developing countries such as Peru. The objective of this study was to compare BMI, adherence to a healthy lifestyle, and breakfast consumption patterns among Peruvian university students of four religious denominations: SDA, Catholic, Baptist, and Evangelical.

## 2. Materials and Methods

### 2.1. Design and Participants

During the first academic semester of the year 2021, between March and April, an observational, analytical, and cross-sectional study was conducted at a private university with campuses in the cities of Lima (Coast), Juliaca (Highlands), and Tarapoto (Jungle), Peru. The study participants were students enrolled in the 2021-I academic year, studying from the first to the seventh year in the faculties of health sciences, business sciences, education sciences and humanities, and engineering and architecture. This study excluded students who did not provide electronic informed consent, those under 18 years of age, and those who did not fully answer all questions in the questionnaire.

The data were collected via nonprobability convenience sampling using an online survey, available on the university’s website. Considering the context of multiple regression, the required sample size was determined using the Soper Free Statistic Calculators version 4.0 software, with the following parameters: an expected effect size (*f*^2^) of 0.02, a desired statistical power level of 0.80, a number of predictors of three variables, and a significance level of 0.05. The choice of an effect size (*f*^2^) of 0.02 was based on Cohen’s guidelines, which classify this size as small, suitable for detecting small but significant associations in complex and multifactorial studies [28]. This resulted in a sample size of 543 students. However, 4557 participants were surveyed.

### 2.2. Ethical Aspects

Before beginning this study, approval from the Ethics Committee of the Faculty of Health Sciences of the Universidad Peruana Unión was obtained (approval code: 0037-2020/UPeU/FCS/CIISA, 12 November 2020). All participants provided their informed electronic consent before participating in this study. The procedures used were in accordance with the guidelines and regulations set forth in the Declaration of Helsinki, thus ensuring that the methods utilized adhered to established ethical principles for research involving human subjects.

### 2.3. Study Variables

#### 2.3.1. BMI

The weight and height of the participants were self-reported. Subsequently, the BMI was calculated as weight (in kilograms) divided by height (in meters) squared (kg/m^2^). The BMI was evaluated according to the criteria established by the Peruvian Ministry of Health, as outlined in the Technical Guide for the Anthropometric Nutritional Assessment of Adults [29]. Participants were classified according to their BMI into the following categories: (a) thin, with a BMI less than to 18.5 kg/m^2^; (b) normal weight, with a BMI of 18.5 to 24.9 kg/m^2^; (c) overweight, with a BMI of 25.0 to 29.9 kg/m^2^; and (d) obese, with a BMI of 30 kg/m^2^ or more [30].

#### 2.3.2. Adherence to a Healthy Lifestyle

Assessments of participant lifestyle information were conducted using the Diet and Healthy Lifestyle Scale (DEVS) [31], which was developed following the recommendations of the guidelines for healthy vegetarian diets and lifestyles proposed by the Department of Nutrition, School of Public Health, Loma Linda University [32,33]. The instrument consists of 14 items that generate information related to the following five domains: (1) plant-based diets, (2) consumption of animal foods, (3) regular physical exercise, (4) adequate water intake, and (5) moderate exposure to sunlight. Each question presents three potential responses. The total score of the instrument, which ranges from 0 to 14 points, is calculated by summing the scores assigned to each item, with each category of the item receiving a score of 0, 0.5, or 1 point. Participants receive a score of 1 point when they report consuming ≥6 daily servings of whole grains, ≥3 daily servings of legumes, ≥8 daily servings of vegetables, ≥4 daily servings of fruits, ≥1.5 daily servings of nuts and seeds, ≤2 daily servings of vegetable oils, and 0 daily servings of dairy products and eggs [34]. Higher scores reflect greater adherence to healthy lifestyle habits. The reliability of the instrument was assessed using several internal consistency coefficients. The results showed high reliability with an ordinal alpha (α) of 0.80, an omega coefficient (ω) of 0.83, and an H coefficient of 0.84 [31].

#### 2.3.3. Breakfast Consumption

To assess the frequency with which the participants ate breakfast, the following question was used: “How often do you eat breakfast per week?”, which has been used in previous research [35,36,37]. Participants were asked to indicate, using a numerical response on a scale of 0 to 7, the number of days a week on which they typically ate breakfast. The responses indicated that the frequency of breakfast consumption could be classified into three categories: those who ate breakfast “rarely or never” (0 to 2 days a week), the “occasional” group (3 to 5 days a week), and those who ate breakfast “regularly” (6 to 7 days a week). This methodology is based on previous research that used a similar classification to assess breakfast habits [36,37].

#### 2.3.4. Religious Affiliation

The collection of information on the religious affiliation of the participants was carried out through the following question: “To which religious denomination do you belong?” Participants were able to choose from several pre-determined options that included the major religious denominations represented at the university (SDA, Baptist, Catholic, and Evangelical). This question allowed us to classify participants according to their religious affiliation and analyze how this variable could influence BMI, lifestyle adherence, and breakfast consumption. Information was also collected on other categories relevant to this study, such as age, sex, place of origin, place of residence, marital status, field of study, and others.

### 2.4. Statistical Analysis

The variables were described using mean and standard deviation for the numerical variables and absolute and relative frequencies for the categorical variables. General characteristics, anthropometric parameters, lifestyle, and breakfast consumption were compared according to religious affiliation. The evaluation of statistically significant differences (*p* < 0.05) was conducted using the ANOVA for numerical variables and the chi-square test for categorical variables. Using linear regression models (beta), we evaluated whether the average lifestyle score and BMI were associated with religious affiliation. Poisson regression models with robust variance were also used to calculate prevalence ratios (PRs) with 95% confidence intervals (95% CIs) to assess the association between religious affiliation and regular breakfast consumption. Statistical analysis was performed with R version 4.3.2.3.

## 3. Results

Sociodemographic data of university students according to religious affiliation are presented in Table 1. A total of 4557 students participated in this study, of which 54.8% (*N* = 2497) identified themselves as members of the SDA Church. Approximately 54.0% of the participants were female. Catholic students had a higher representation from the Sierra (71.7%) and a higher proportion of urban residents (75.3%) compared to other religious groups (*p* < 0.001). Most of the students were single (94.2%). Parents of Adventist students had a basic education mainly (60.2%, *p* < 0.001). The SDA Church students had a higher representation on the health sciences faculty (34.4%) compared to the other groups (*p* < 0.001).

Table 2 presents a comparative analysis of anthropometric characteristics, adherence to a healthy lifestyle, and breakfast consumption among Peruvian university students of different religious affiliations. There was no significant difference in BMI scores; in fact, both Catholic and Adventist students reported healthy scores for this indicator of nutritional status (*p* > 0.05). However, in terms of BMI categories, overweight/obesity was less prevalent among SDA students compared to other denominations (*p* = 0.024). The data indicated that more than half of the participants exhibited healthy breakfast consumption patterns, with a higher prevalence observed among the SDA students.

In Table 3, after adjustment for the variables, the Baptist (B = 0.44, 95% CI: 0.10 to 0.78; *p* = 0.011), Catholic (B = 0.3, 95% CI: 0.12 to 0.47; *p* = 0.001), and Evangelical (B = 0.32, 95% CI: 0.09 to 0.64; *p* = 0.014) students had a significantly higher BMI compared to Adventists. On the other hand, Baptist students (B = −0.2, 95% CI: −0.37 to −0.05; *p* = 0.017) and Evangelical students (B = −0.13, 95% CI: −0.28 to −0.03; *p* = 0.012) had a lower mean score on the healthy lifestyle measure compared to SDA students. Additionally, Baptist students (PR = −0.32, 95% CI: −0.92 to −0.12; *p* = 0.035) and Catholic (PR = −0.3, 95% CI: −0.99 to −0.19; *p* = 0.016) students showed a lower probability of eating breakfast regularly compared to SDA students. The coefficients in the Poisson regression are presented as PR, which, in this context, determine the relative probability of an event (regular breakfast consumption) occurring in different religious groups compared to the reference group (SDA). Negative coefficients indicate a lower relative probability of regular breakfast consumption.

## 4. Discussion

BMI, adherence to a healthy lifestyle, and regular breakfast consumption are critical factors that can influence the health and well-being of university students. These young people face many challenges, including academic pressures, the transition to an environment of greater independence, and social influences that can affect their lifestyles, which can have long-term effects on their health. In this context, religious affiliation may be important in shaping these life practices. In fact, for some religious denominations, religious beliefs and practices often include lifestyle recommendations that can promote healthy behaviors. This study aims to fill this literature gap by providing data on how religious affiliation is associated with BMI, adherence to a healthy lifestyle, and breakfast consumption among Peruvian university students.

The main findings of this study indicate that (a) compared to SDA students, Baptist, Catholic, and Evangelical students had a significantly higher BMI; (b) similarly, Baptist and Evangelical students had a lower mean score on the measure of healthy lifestyle compared to SDA students; finally, (c) Baptist and Catholic students showed a lower likelihood of eating breakfast regularly compared to SDA students.

BMI. An elevated BMI in university students can have serious implications for physical and mental health, regardless of religious affiliation. Overweight and obesity in college not only increase the risk of developing chronic diseases such as type 2 diabetes, hypertension, and cardiovascular disease [38], but can also lead to higher levels of stress, anxiety, and depression [39] which, in turn, can negatively affect student academic performance and quality of life [40]. In the current study, overweight/obesity was less prevalent among SDA students compared to other denominations. These findings could be due, although partially, to the fact that SDA students had a higher mean adherence to a healthy lifestyle and demonstrated a higher frequency of breakfast consumption than the other denominational groups evaluated in this study. In addition, it is important to mention that many members of the SDA Church often consume their last two daily meals in the evening, which results in an extended overnight fast that can contribute to weight loss [41].

Likewise, a study conducted in Poland finds differences in Catholic BMI when comparing SDA, reporting that almost twice as many Catholics were obese [42]. Furthermore, research conducted among various Christian denominations indicated that people who identify as Baptists or Evangelical Christians are more likely to be obese [7,20]. Similarly, a study conducted on Australian adults compared the body weight of SDA with patients in a general practice clinic and volunteers. The results indicated that SDA exhibited significantly lower body weights compared to the other two groups [43]. These findings are consistent with those of another study, which indicated that Adventists had a lower prevalence of obesity compared to non-Adventists [44]. In a longitudinal intervention study, non-SDA were found to experience a greater reduction in weight and BMI compared to SDA over a 30-day follow-up period; specifically, the mean change in BMI was −1.0 in SDA versus −0.93 in non-ADS [44]. The results of the present study highlight the need to incorporate religious factors into the development of public health interventions designed to address the problem of overweight and obesity among university students.

Adherence to a healthy lifestyle. Baptist and Evangelical students exhibited a lower level of adherence to the healthy lifestyle measure compared to SDA students. This finding may be interpreted as an indication that the dietary and lifestyle practices taught by the SDA are more stringent and widely followed by its members. In this study, dietary aspects, along with physical activity, water consumption, and sunlight exposure, were among the lifestyles evaluated. Regarding dietary practices, SDA are distinguished by their adhesion to a plant-based diet, abstinence from alcohol and tobacco, and promotion of regular physical activity. Furthermore, they advocate for adherence to kosher laws [45]. A study comparing alcohol consumption and smoking between SDA and Catholics found that 100% of the SDA surveyed abstained from the consumption of these substances compared to Catholics [42]. Furthermore, the same study indicated that the SDA under examination consumed a higher proportion of nutritionally beneficial foods, including whole-grain bread, skim/low-fat dairy products, brown rice, pasta, multigrain semolina, multigrain and wheat flakes, white fish, vegetable fats, and legumes [42].

The SDA doctrine posits that the human body is the physical dwelling place of the Holy Spirit. Therefore, it is held that any condition affecting the body will also affect the mind. Furthermore, it is believed that any occurrence within the body will facilitate or hinder communication with celestial beings [46]. The approach of the SDA Church to health projects is twofold. First, it develops health institutions, including clinics, hospitals, and centers for healthy living, with a focus on prevention. Second, it provides educational resources, such as courses, seminars, conferences, workshops, and practical events, to teach parishioners and the general public how to improve their quality of life [24]. The foundation of these health initiatives is rooted in the concept of eight natural remedies, which encompass clean air, rest, exercise, sunlight, water, nutrition, temperance, and hope in God [47]. These practices are deeply embedded in the religious beliefs and philosophy of the Church and are promoted through its community, educational, and health institutions [19,24]. These health practices may explain the observed differences in BMI, prevalence of chronic diseases such as diabetes and hypertension, and life expectancy between SDA and other religious groups [48]. The success of the SDA Church in maintaining a healthy lifestyle can serve as a model for other religious and secular communities, demonstrating how beliefs and community support can be leveraged to improve public health.

Although our findings indicate that SDA students are less likely to have excess body weight and adhere to a healthier lifestyle, it is important to note that these results should not be interpreted as evidence of the superiority of one religious group over another. A variety of factors, including cultural practices, socioeconomic status, and the availability of support networks, can exert a considerable influence on health behaviors and outcomes within the university context.

Regular consumption of breakfast. Baptist and Catholic students showed a lower likelihood of eating breakfast regularly compared to SDA students. However, in the comparative analysis, using the chi-square test of independence, healthy patterns of breakfast consumption were observed in the majority of participants. This finding suggests that, although SDA tend to have healthy dietary behaviors, students of other religious denominations have also demonstrated positive breakfast consumption habits. This is evidenced by the findings of a study by Majda et al. [42], where only 10.2% of the SDA reported eating breakfast regularly, compared to Catholics (88.8%). These results are surprising given that SDA are known for their good eating habits. It is therefore important to recognize that different religious denominations may have specific strengths in relation to certain health practices. Diversity in health behaviors underscores the need for personalized and respectful approaches that consider the unique practices and beliefs of each religious group.

However, the dietary teachings of the SDA emphasize not only the type of food consumed, but also the quantity and frequency [41]. Promoting two balanced meals per day may be a contributing factor to observed patterns of breakfast consumption.

Furthermore, unlike the study by Majda et al. [42], our study was conducted in university students, since, in the context of university students, dietary patterns can vary significantly due to academic load, lifestyle, and personal preferences. The discrepancy in the frequency of breakfast consumption between SDA and Catholic students in this context may be indicative of individual adaptations to the demands of university life, rather than a reflection of strict adherence to religious teachings. Students who regularly eat breakfast tend to have better academic results, greater concentration and memory capacity, and superior cognitive tasks performance compared to those who skip this meal [1]. Eating carbohydrates, proteins, and healthy fats at breakfast helps maintain stable blood glucose levels, which is essential for optimal brain function [49]. These benefits of breakfast are particularly important in the university setting, where students face significant cognitive and physical demands.

### 4.1. Public Health Implications

BMI is a key indicator of the risk of developing non-communicable diseases; therefore, the adoption of healthy lifestyle patterns promoted by the SDA Church, including a plant-based diet, regular physical activity, adequate rest, and abstinence from harmful substances such as alcohol and tobacco, could be a strategy to reduce the prevalence of obesity and its associated complications in university students and the general population. In addition, understanding the various religious dietary norms and their possible impact on BMI can guide nutritionists and other health professionals in the care of university students, without interfering in any way with one’s own religious convictions [45]. 

Also, given the established link between regular breakfast consumption and a variety of benefits, including enhanced academic performance, increased concentration, and a reduced risk of developing metabolic diseases, public health initiatives that encourage regular and healthy breakfasts, particularly among youth and college students, could have a considerable impact on their health and academic performance. Educational programs in schools and universities that emphasize the importance of breakfast could help establish healthy habits from an early age. Similarly, public health policies could focus on educating the population about the benefits of a diet rich in fruits, vegetables, whole grains, and nuts, as well as encouraging regular physical activity and reducing the consumption of ultra-processed foods.

### 4.2. Limitations and Future Considerations

Although this study included a substantial number of participants, it is essential to consider the potential limitations when interpreting the results. The weight and height of the participants were self-reported. However, some studies have indicated that BMI based on self-reported data can also classify the majority of the population into the correct BMI categories [5]. Nevertheless, self-reported data are subject to bias and inaccuracies, and direct measurements would provide more reliable data. Furthermore, the data collection period spanned a single academic semester, which may not be representative of long-term changes in lifestyle and BMI. This limited timeframe does not account for the seasonal variability in food consumption and lifestyle habits, which could influence the results.

Additionally, this study was conducted at a single private university, which most certainly does not represent the entire Peruvian population. This limits the generalizability of the findings, as university culture and religious practices can exhibit considerable variation between different institutions and geographic areas. It is notable that university culture and religious practices can exhibit considerable variation between different institutions and geographic areas; this university has campuses located in the three geographic regions of Peru: the coastal area, the highlands, and the jungle. Choosing a private university may also introduce bias, as the student population may have different socioeconomic backgrounds compared to those attending public universities. The private setting is likely to reflect the upper-middle socioeconomic status of students, excluding those from lower socioeconomic backgrounds who attend public institutions or cannot afford higher education.

On the other hand, convenience sampling was used instead of random sampling, which can potentially compromise the validity of the data.

Although several religious denominations were analyzed, the categorization may not capture the diversity within each religious group; there may be significant differences in practices and beliefs within the same denominations that were not addressed in this study. As this study was conducted during the ongoing pandemic, it is possible that the lifestyle habits and dietary practices of the student cohort may have been influenced by the restrictions and changes in daily life that resulted from the pandemic. Such circumstances may have influenced the results and may not necessarily reflect the typical practices of students in non-pandemic times.

Future studies should consider including a more diverse sample from multiple universities, including public institutions, and extend the data collection period to cover multiple semesters to better capture seasonal variability and long-term trends in lifestyle and dietary habits.

It is important to note that although our results show significant differences among the religious groups studied, we do not intend to suggest that one religious affiliation is superior to another in promoting healthy habits. The results should be interpreted with caution, taking into account the limitations of this study and the specific cultural context of the sample. Our purpose is to contribute to the understanding of how different factors, including religiosity, may be associated with health behaviors. Future research should explore these associations in different contexts and with more diverse samples to confirm and extend our findings. In addition, the results should be considered as a basis for developing public health interventions that are culturally sensitive and respectful of the beliefs and practices of all religious groups.

## 5. Conclusions

The findings of this cross-sectional study demonstrate a significant correlation between religious affiliation and several health-related behaviors among Peruvian university students, including BMI, adherence to healthy lifestyles, and breakfast consumption. Specifically, SDA students had lower BMI and higher healthy lifestyle scores and were more likely to eat breakfast regularly compared to Baptist, Catholic, and Evangelical students. These findings suggest that specific diet practices and teachings for certain religions, such as those of the SDA Church, could have a positive impact on the health of university youth. Therefore, health professionals should consider these findings when designing and implementing health promotion programs that are culturally sensitive and respectful of the beliefs and practices of all religious groups in the university setting.

## Figures and Tables

**Table 1:** General characteristics according to the religious affiliation of university students (*N* = 4557).

Characteristics		Religious Affiliation				
	All	Adventist	Baptist	Catholic	Evangelical	*p*-Value *
	*N* = 4557	*N* = 2497	*N* = 255	*N* = 1522	*N* = 283	
		(54.80%)	(5.60%)	(33.40%)	(6.20%)	
Age (M ± SD)	21.4 ± 3.4	21.7 ± 3.5	21.7 ± 3.4	20.8 ± 3.1	21.6 ± 3.7	<0.001
Age group (years)						<0.001
18	738	352 (14.1%)	45 (17.6%)	301 (19.8%)	40 (14.1%)	
19–24	3142	1718 (68.8%)	163 (63.8%)	1061 (69.7%)	200 (70.7%)	
>24	677	427 (17.1%)	47 (18.6%)	160 (10.5%)	43 (15.2%)	
Sex						0.285
Female	2478	1348 (54.0%)	134 (52.7%)	851 (55.9%)	145 (51.3%)	
Male	2079	1149 (46.0%)	121 (47.3%)	671 (44.1%)	138 (48.7%)	
Provenance						<0.001
Coast	998	746 (29.9%)	26 (10.3%)	142 (9.3%)	84 (29.8%)	
Jungle	964	485 (19.4%)	97 (38.1%)	283 (18.6%)	99 (35.0%)	
Highlands	2488	1173 (47.0%)	131 (51.1%)	1090 (71.7%)	94 (33.3%)	
Foreign	107	93 (3.7%)	1 (0.5%)	7 (0.5%)	6 (2.0%)	
Residence						<0.001
Rural	1293	779 (31.2%)	67 (26.2%)	376 (24.7%)	71 (25.2%)	
Urban	3264	1718 (68.8%)	188 (73.8%)	1146 (75.3%)	212 (74.8%)	
Marital status						0.012
Married	257	145 (5.8%)	21 (8.4%)	72 (4.7%)	19 (6.8%)	
Singles	4300	2352 (94.2%)	234 (91.6%)	1450 (95.3%)	264 (93.2%)	
Parent’s education						<0.001
Basic	2608	1503 (60.2%)	132 (51.6%)	828 (54.4%)	145 (51.3%)	
Technical	811	420 (16.8%)	44 (17.3%)	286 (18.8%)	61 (21.3%)	
Undergraduate	702	357 (14.3%)	51 (20.0%)	257 (16.9%)	37 (13.2%)	
Postgraduate	436	217 (8.7%)	28 (11.1%)	151 (9.9%)	40 (14.2%)	
Faculty						<0.001
Health	1465	859 (34.4%)	74 (28.9%)	460 (30.2%)	72 (25.4%)	
Business	1200	539 (21.6%)	81 (31.6%)	492 (32.3%)	88 (31.1%)	
Humanities and Education	489	357 (14.3%)	19 (7.8%)	87 (5.7%)	26 (9.3%)	
Engineering and Architecture	1403	742 (29.7%)	81 (31.6%)	483 (31.8%)	97 (34.2%)	

Note. * Statistical significance by ANOVA or chi-square test of independence.

**Table 2:** Anthropometric parameters, adherence to a healthy lifestyle, and breakfast consumption according to the religious affiliation of the university students.

Characteristics		Religious Affiliation				
	All	Adventist	Baptist	Catholic	Evangelical	*p*-Value *
	*N* = 4557	*N* = 2497	*N* = 255	*N* = 1522	*N* = 283	
		(54.80%)	(5.60%)	(33.40%)	(6.20%)	
Weight (M ± SD)	63 ± 11	61 ± 11	64 ± 11	63 ± 10	64 ± 12	<0.001
Height (M ± SD)	1.62 ± 0.08	1.62 ± 0.08	1.63 ± 0.09	1.63 ± 0.08	1.63 ± 0.09	<0.001
BMI (M ± SD)	23.8 ± 3.2	23.7 ± 3.2	24.0 ± 3.4	23.7 ± 3.1	24.0 ± 3.7	0.175
BMI categorized						0.024
Underweight	118 (2.6%)	61 (2.4%)	10 (4.1%)	41 (2.7%)	6 (2.2%)	
Normal	3136 (68.8%)	1740 (69.7%)	159 (62.4%)	1044 (68.6%)	193 (68.5%)	
Overweight	1113 (24.4)	594 (23.8%)	74 (28.9%)	381 (25.0%)	64 (22.5%)	
Obesity	190 (4.2%)	103 (4.1%)	12 (4.6%)	56 (3.7%)	19 (6.8%)	
Adherence to a healthy lifestyle (M ± SD)	6.51 ± 1.50	7.55 ± 1.53	6.35 ± 1.51	6.50 ± 1.46	6.42 ± 1.53	0.048
Breakfast consumption						0.039
Rarely or never (0–2 days)	1189 (26.1%)	629 (25.2%)	70 (27.3%)	408 (26.8%)	82 (29.1%)	
Some days (3–5 days)	752 (16.5%)	410 (16.4%)	46 (18.1%)	256 (16.8%)	40 (13.9%)	
Regularly (6–7 days)	2616 (57.4%)	1458 (58.4%)	139 (54.6%)	858 (56.4%)	161 (57.0%)	

Note. * Statistical significance by ANOVA or chi-square test of independence; BMI: body mass index.

**Table 3:** Simple and multiple regression models between religion, BMI, adherence to a lifestyle, and breakfast consumption.

	Simple Regression			Multiple Regression *		
	BMI					
Religious affiliation	B	95% CI	*p*	B	95% CI	*p*
Adventist	Ref.			Ref.		
Baptist	0.33	−0.02–0.67	0.061	0.44	0.10–0.78	0.011
Catholic	0.05	−0.12–0.22	0.576	0.30	0.12–0.47	0.001
Evangelical	0.30	−0.03–0.62	0.077	0.32	0.09–0.64	0.014
	Adherence to a healthy lifestyle					
Religious affiliation	B	95% CI	*p*	B	95% CI	*p*
Adventist	Ref.			Ref.		
Baptist	−0.20	−0.36–−0.04	0.014	−0.20	−0.37–−0.05	0.017
Catholic	−0.05	−0.13–0.03	0.225	−0.05	−0.15–0.01	0.256
Evangelical	−0.13	−0.31–−0.03	0.003	−0.13	−0.28–−0.03	0.012
	Regular breakfast consumption					
Religious affiliation	PR	95% CI	*p*	PR	95% CI	*p*
Adventist	Ref.			Ref.		
Baptist	−0.34	−0.95–−0.15	0.036	−0.32	−0.92–−0.12	0.035
Catholic	−0.31	−0.99–−0.18	0.017	−0.30	−0.99–−0.19	0.016
Evangelical	1.07	0.98–1.17	0.126	1.05	0.96–1.14	0.299

Note. PR: prevalence ratio; B: regression coefficient; 95% CI: 95% confidence interval; BMI: body mass index. Linear regression was used for the BMI and adherence to a lifestyle score, and Poisson regression with robust variance was used for the variable regular breakfast consumption. * Adjusted for age group, residence, provenance, sex, origin, marital status, and father’s education.

## Data Availability

The data presented in this study are available on request from the corresponding author. The data are not publicly available, due to privacy and ethical concerns.
